# Non-Trileaflet Aortic Valve Aortopathies

**DOI:** 10.3390/life15050713

**Published:** 2025-04-28

**Authors:** Abdelrahman Ahmed, Tom Kai Ming Wang

**Affiliations:** Section of Cardiovascular Imaging, Department of Cardiovascular Medicine, Heart, Vascular, and Thoracic Institute, Cleveland Clinic, 9500 Euclid Avenue, Main Campus, J1-5, Cleveland, OH 44195, USA; ahmeda19@ccf.org

**Keywords:** aortic aneurysms, bicuspid aortic valve, unicuspid aortic valve, quadricuspid aortic valve

## Abstract

The incidence of thoracic aortic aneurysms (TAAs) is approximately 10.4 cases per 100,000 person-years. Although most cases of TAA are caused by degenerative disease, associated aortic valve abnormalities have been heavily linked to this condition. These include unicuspid, bicuspid and quadricuspid aortic valves. These non-tricuspid aortic valves occur sporadically but can occur in familial clusters with variable penetrance. The presence of non-tricuspid aortic valves has significant implications for patients, as they become prone to valvular dysfunction and aortic dissection. Therefore, understanding of the pathophysiology and natural history of this condition is imperative for early diagnosis, regular surveillance and timely intervention. In this review article, we discuss the normal anatomy of the aortic valve, non-tricuspid aortic valves and their association with TAAs. We also highlight the role of various cardiac imaging modalities in the management of affected patients.

## 1. Introduction

Thoracic aortic aneurysms (TAAs) are characterized by the dilation of one or more segments of the aorta above the diaphragm. The pathogenesis of TAA is multifactorial, involving a complex interplay of genetic, environmental and hemodynamic factors that contribute to the progressive weakening of the aortic wall, ultimately leading to dilation. Once the aorta becomes dilated, patients are at increased risk for life-threatening complications, including wall rupture, aortic dissection and sudden death. Aortic dissection can extend proximally, potentially involving the aortic valve (AV) and resulting in valve dysfunction [1].

In parallel, primary abnormalities in AV structure, particularly deviations from the normal trileaflet configuration, have been linked to aortic root disease and poor prognosis. The relationship between non-tricuspid valve anatomy and TAA remains poorly understood, with debate surrounding whether the association is primarily genetic or the result of biomechanical stress on the proximal aorta [2].

This review aims to summarize the current literature on the incidence of non-trileaflet aortic valves, aortic aneurysms and the link between these conditions. We also highlight the critical role of multimodality imaging in the diagnosis and management of these complex patients. Finally, we briefly describe treatment options for patients with TAA, including medical therapies, and the important role of open surgical and endovascular repairs.

## 2. Anatomy of the Aorta

The thoracic aorta refers to the portion of the aorta located above the diaphragm. It is divided into five distinct segments: the aortic root, ascending aorta, aortic arch, isthmus and descending aorta. The aortic root is situated between the left ventricular outflow tract (LVOT) and the ascending aorta. It is anatomically related to the AV, being bordered proximally by the basal attachment of the AV and distally by the peripheral attachment of the aortic cusps to the sinotubular junction (STJ). The abdominal aorta refers to the portion of the aorta below the diaphragm that terminates at the aortic bifurcation into the common iliac arteries, usually at the level of the fourth lumbar vertebra [2].

### 2.1. Anatomy of the Aortic Valve and Root

The AV is positioned between the left ventricular outflow tract (LVOT) and the ascending aorta. It is centrally located along the antero-superior surface of the heart, with the pulmonary valve lying anteriorly. Structurally, the AV consists of four components: the annulus, the cusps, the commissures and the interleaflet triangles. The aortic annulus, a crown-like structure, serves to support the leaflets and facilitate their suspension. The aortic cusps, or leaflets, are bounded distally by the sinotubular junction on the aortic side and extend proximally through the ventriculo-arterial junction into the left ventricle. The basal portions of the cusps converge to form the virtual annular ring. The AV comprises three semilunar leaflets, each contributing to the valve’s function. During valve closure, the leaflets come together at the commissures along the line of apposition. The interleaflet triangles are fibrous, wedge-shaped structures located within the LVOT, extending from the commissures to the sinotubular junction. The aortic root, which lies between the left ventricular outflow tract and the ascending aorta, includes the AV leaflets, the sinuses of Valsalva, the commissures and the interleaflet triangles.

Due to the close anatomical relationship between the AV and the aortic root, any pathology affecting the root may influence the structure and function of the AV, and vice versa. For example, non-trileaflet AVs can affect the nature of the jet and blood flow pattern into the proximal aorta, as evidenced by 4-D flow cardiac magnetic resonance (CMR) imaging. Compared to normal flow in trileaflet AVs, the jet in non-trileaflet AVs has several altered physical properties, including jet angle, wall shear stress, in-plane rotational flow and systolic retrograde flow, which contribute to an increased incidence of TAAs [3].

### 2.2. Bicuspid Aortic Valve: Prevalence, Presentation and Complications

A bicuspid aortic valve (BAV) is the most common congenital heart defect, affecting approximately 0.5% to 2% of the general population, as identified in autopsy and echocardiographic studies [4]. It has a higher prevalence in males, with a male-to-female ratio ranging from 2:1 to 4:1 [5,6,7]. Sillesen et al. reported that the prevalence of BAV in newborns is 0.77%, with associated aortopathy commonly observed, suggesting that BAV may develop as a fetal malformation [5]. The genetics of BAV are complex. Current data suggest that BAV may be inherited in an autosomal dominant fashion but with incomplete penetrance and variable phenotypic expression. It presents as a feature in various clinical syndromes including Turner Syndrome, Loeys–Dietz Syndrome (LDS), Down syndrome and velocardiofacial syndrome. It is hypothesized that copy number reduction in X chromosome genes may be implicated in the development of BAV. However, most known genetic mutations to date do not explain most cases of non-syndromic BAV. Therefore, more research is needed to fully understand the complex genetic pathways implicated in the development of BAV [8].

**Figure 1:**
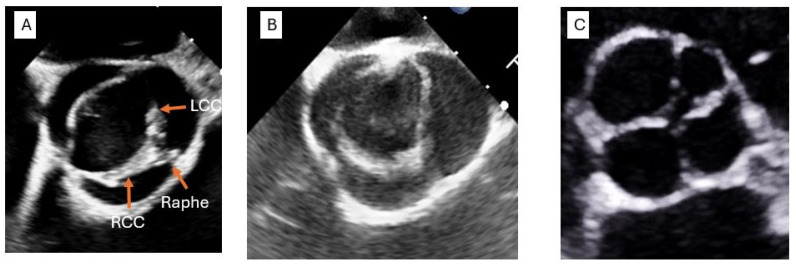
Examples of non-tricuspid aortic valve morphologies on echocardiography. (**A**) Short-axis view of the AV by TEE showing BAV with a raphe between the RCC and LCC. (**B**) Short-axis view of the AV by TEE of a UAV. (**C**) TTE image of a Type C QAV characterized by two larger equally sized cusps and two smaller equally sized cusps. RCC: Right Coronary Cusp; LCC: Left Coronary Cusp.

BAV should be suspected and screened for in patients with coarctation of the aorta, TAAs or calcifications of the aortic valve at a young age and in patients with non-central coaptation of the leaflets as seen in the parasternal long-axis view (PLAX) on echocardiography. BAV can present with several phenotypes, with cusp malformation ranging from the absence of commissures to the under-development of one or two cusps. Figure 1 compares AV morphology in patients with BAV to that in other non-trileaflet AV morphologies. Numerous nomenclature systems have been proposed to describe BAVs in the literature. The widely used Sievers classification, which employs a numeric system to characterize BAV morphologies, has been criticized for its lack of linguistic intuitiveness and its failure to account for certain BAV phenotypes [9]. For instance, Sievers Type 2 BAV is, in fact, a unicuspid aortic valve (UAV). Additionally, the Sievers system does not incorporate aortopathy morphology—an important consideration in the evaluation of patients with BAV. In response to these limitations, a recent expert consensus document proposed an imaging-based nomenclature that classifies BAVs according to the degree of leaflet fusion, the number of sinuses and the presence or absence of raphes [10]. The primary complications linked to BAV include valvular dysfunction, aortic aneurysm, infective endocarditis and aortic dissection [7]. In adults, BAV is typically diagnosed when symptoms from one of these complications emerge. In children, it is often detected through the screening of family members when a relative has already been diagnosed with BAV. The age-adjusted relative risk of developing an aortic aneurysm is 80 times higher in patients with BAV than in the general population. Although relatively uncommon, the age-adjusted relative risk (8.4) of aortic dissection in patients with BAV is significantly higher than that in people without BAV [11,12].

### 2.3. Unicuspid Aortic Valve

UAV is a rare congenital anomaly characterized by abnormal valve morphology and is often poorly understood in terms of its natural history. The diagnostic criteria for UAV generally include at least two of the following three findings: (1) an obtuse angle between the fused cusps at the commissure, (2) the absence of a distinct separating plane between the fused cusps and (3) the presence of a raphe at the point of commissural fusion. These features collectively impart an exclamation point-like appearance to the valve [13].

Patients with UAV experience similar complications to those with BAV, including valvular dysfunction, calcification, aortic aneurysm and dissection. However, these complications often manifest earlier and progress more rapidly in UAV [14]. Aortic stenosis, with or without aortic regurgitation (AR), is the most common form of valvular dysfunction in UAV, while isolated AR is relatively uncommon. Symptomatic presentation typically occurs in the third or fourth decade of life, and surgical intervention is often required to alleviate symptoms [13,15]. A study of 75 pathologically confirmed UAV patients followed at Mayo Clinic revealed that three-quarters of these individuals had dilation of the aortic root or ascending aorta at the time of diagnosis, with a mean diameter of 4.4 ± 2.1 mm. Notably, one-third of patients exhibited a ≥2 mm increase in aortic diameter over a 10-year follow-up period. The risk of aortic dissection in patients with UAV remains extremely low, although it remains five to eight times higher than that in the general population [15]. Overall, these findings highlight the significant association between UAV and aortopathy.

### 2.4. Quadricuspid Aortic Valve

Quadricuspid aortic valves (QAVs) are exceedingly rare, with an estimated incidence of less than 0.05% [16]. According to the classification system proposed by Hurwitz and Roberts, QAVs are divided into six types: type A, four relatively equally sized cusps; type B, three equal-sized cusps and one smaller cusp; type C, two larger equally sized cusps and two smaller equally sized cusps; type D, one larger cusp, two intermediately sized cusps and one smaller cusp; type E, three relatively equal-sized cusps and one larger cusp; and type F, two relatively equally sized larger cusps and two unequal smaller cusps [17].

A single-center study involving 50 patients with QAV revealed that aortic aneurysm was present in approximately one-third of the patients at the time of diagnosis. The dilations were equally common in the aortic root and ascending aorta. The degree of aortic dilation was generally described as mild (<5 mm above the upper limit of the reference range) in 79% of the patients with aneurysm and as moderate (5 to 10 mm above the upper limit of the reference range or between 45 and 50 mm) in 21% of the patients. Furthermore, aortic dilation in this cohort was notably associated with more moderate to severe aortic regurgitation compared to that in patients without dilation. However, the rate of aneurysmal growth was extremely slow in most cases, with patients rarely requiring surgical intervention to address the aneurysm. Therefore, valve dysfunction remains the primary morbidity in patients with QAV. Despite these findings, the presence of aortic dilation was associated with worse survival free of AV surgery or death [18].

### 2.5. Definition and Incidence of Thoracic Aortic Aneurysm

An artery is labeled as dilated if its diameter has increased to 1.5 times the normal expected diameter. This definition correlates well with descending aortic and abdominal aneurysms but poses significant challenges in the diagnosis of aortic root and ascending aortic aneurysms [2]. For example, if this definition is applied to a 45-year-old male, the aortic root would have to measure 5.25 cm before it is diagnosed as aneurysmal since the expected normal aortic diameter in this demographic is ~3.5 cm. This would obviously result in delayed diagnosis and care for such patient with increased risk of aneurysm rupture and death. Indeed, an analysis of thousands of patients enrolled in the MESA study showed that among patients without known cardiovascular disease, the mean size of the normal aorta was 3.2 cm, and the maximum ascending aorta size was 5.0 cm in men and 4.9 cm in women. The authors also demonstrated that the relative risk of aortic dissection in patients with an aortic size ≥ 4.5 cm compared to those with an aortic size of <3.4 cm was a massive 6305. Therefore, it is prudent to classify patients with an ascending aortic size of 4.0–4.4 cm as having dilated aortas, while patients with aortic sizes ≥ 4.5 cm can be labeled as having aortic aneurysms [19].

The incidence of thoracic aortic aneurysm (TAA) is approximately 10.4 cases per 100,000 person-years [20]. It is slightly more common in males than in females, with a male-to-female ratio of 1.7:1. These aneurysms most frequently occur in the ascending thoracic aorta, often involving the aortic arch (40%), while 31% affect the descending aorta. Both the ascending and descending aorta are involved in 29% of cases [20,21]. Most TAAs are idiopathic. Atherosclerosis is thought to be the main driving factor of descending aortic aneurysms, while ascending aortic aneurysms are linked to various risk factors, including genetic defects, abnormal aortic valve morphology and hemodynamic factors [21]. TAAs are associated with increased morbidity and mortality, primarily due to the risk of aneurysm rupture. The cumulative 5-year probability of TAA is 20% [21].

### 2.6. Pathogenesis of TAA

While atherosclerosis is the primary cause of descending TAAs, the pathophysiologic pathways leading to the development of ascending TAAs are more complex and multifactorial [21]. Several factors, including degenerative changes in the aortic wall, inflammation, systemic hypertension and genetic predisposition, interact to disrupt the normal balance between collagen and elastin in the tunica media. In a healthy aorta, collagen is the predominant structural protein in the extracellular matrix (ECM), providing strength to the arterial wall. Elastin, in contrast, offers the elasticity needed for arterial wall compliance as blood flows from the heart into the ascending aorta. Elastin density is highest at the aortic root and decreases progressively as the aorta transitions into the descending aorta [1]. One of the key histologic features of TAA is the loss of elastin fibers in the tunica media [22]. Excessive activity of matrix metalloproteinases (MMPs), enzymes involved in the degradation of extracellular matrix proteins, has been linked to this loss of elastin. Matrix metalloproteinases are essential for the maintenance of the cell matrix, membrane assembly and endothelial cell differentiation. They are secreted by macrophages and act by degrading various ECM proteins and recruiting other inflammatory cells to the area [23]. Their activity is regulated by tissue inhibitors of metalloproteinases (TIMPs), and an imbalance between MMPs and TIMPs can lead to excessive MMP activity and aortic wall damage. Increased MMP levels and reduced TIMP function have been observed in TAA [24].

A key aspect worth emphasizing is the interplay between AV leaflet anatomy and the pathogenesis of aortic aneurysms. Patients with BAV, UAV or QAV exhibit a higher incidence of aortic aneurysms, a phenomenon attributed to aberrant aortic flow dynamics. These altered hemodynamic patterns result in elevated wall shear stress, a critical factor implicated in vessel wall remodeling and aneurysm formation [25]. The most precise visualization of these hemodynamic changes is achieved through four-dimensional (4-D) cardiac magnetic resonance (CMR) imaging, which enables velocity encoding and facilitates the assessment of time-resolved blood velocity within the aorta [26,27]. In individuals with a normal trileaflet AV, blood flow jets are typically well-centered. Conversely, in non-trileaflet AVs, the eccentricity of these jets contributes to increased wall shear stress in the proximal aorta, acting as a persistent source of mechanical insult. A 4-D flow assessment by CMR allows for the direct assessment of wall shear stress. This repetitive endothelial injury incites inflammation and ultimately predisposes the vessel to regional aortic dilation [3,28].

Aortic aneurysms predispose patients to a spectrum of potentially life-threatening conditions collectively known as acute aortic syndromes (AASs). The primary entities within this category include acute aortic dissection (AAD), intramural hematoma (IMH) and penetrating aortic ulcer (PAU) [2,29]. Among these, AAD is the most prevalent and is characterized by an intimal tear that functions as a one-way valve, permitting blood to dissect into the tunica media. This process creates true and false lumens, with the potential for rapid antegrade or retrograde propagation. Such propagation can precipitate aortic regurgitation, pericardial effusion, myocardial ischemia, stroke or malperfusion syndromes. If left untreated, early mortality can be as high as 2% [30]. IMH is considered a variant of AAD, distinguished by hemorrhage confined within the media in the absence of a detectable false lumen. Diagnosis is best achieved through a dedicated computed tomography angiography (CTA) protocol, incorporating both non-contrast and contrast-enhanced imaging, which typically reveals a crescentic thickening of the aortic wall without intraluminal blood flow [31]. PAU, another AAD variant, primarily affects the intima and arises from the focal disruption of an atherosclerotic plaque, allowing blood to penetrate the media. Contrast-enhanced CTA typically demonstrates PAU as a localized outpouching of the aortic wall that communicates with the lumen [32].

The management of AAS is tailored based on anatomical involvement and clinical severity. AAS affecting the aortic root, arch or ascending aorta (type A) typically necessitates surgical intervention. In contrast, uncomplicated AAS involving the descending aorta (type B) is generally managed with medical therapy, emphasizing blood pressure control and surveillance [33].

## 3. The Role of Multimodality Cardiac Imaging in the Evaluation of Aortic Valve Abnormalities and Aortopathy

The accurate diagnosis of AV abnormalities and associated aortic aneurysms is critical in the management of aortopathy. Advances in cardiac imaging techniques have significantly enhanced our ability to assess both the morphology of the AV and the aortic root, as well as to evaluate the extent of aortic dilation. Current imaging modalities, including echocardiography, computed tomography (CT) and magnetic resonance imaging (MRI), offer distinct advantages in diagnosing non-tricuspid AV aortopathy and identifying potential aneurysmal changes. This section will provide an overview of the key imaging modalities used to evaluate non-tricuspid AV aortopathy. Table 1 describes the main techniques used in each imaging modality for the evaluation of TAA and AV. Table 2 highlights some of the strengths and limitations of each modality.

### 3.1. Transthoracic Echocardiography (TTE)

Transthoracic echocardiography (TTE) remains the first-line imaging modality in the fassessment of AV abnormalities and aortic aneurysms due to its safety profile, accessibility, non-invasive nature and established utility in clinical practice. The PLAX view is particularly useful for evaluating AV morphology, with off-center leaflet coaptation suggesting the presence of a non-tricuspid AV. The aortic root and proximal ascending aorta are well visualized in this view, allowing for accurate measurements of the diameters of key anatomical structures, including the sinuses of Valsalva, sinotubular junction and the proximal ascending aorta. Moreover, the incorporation of color Doppler allows for a detailed assessment of aortic regurgitation, a common concomitant finding in patients with aortic aneurysm.

For the visualization of the mid-ascending aorta, a high-lateral view is often employed. The parasternal short-axis (PSAX) view, obtained by rotating the transducer 90 degrees clockwise from the PLAX position, offers a cross-sectional view of the AV and root, facilitating the identification of raphes or anomalies in leaflet fusion. To assess the aortic arch, the suprasternal view provides optimal visualization, while the descending aorta is typically evaluated using subcostal imaging.

Although TTE provides valuable insights into AV function and root dimensions, its limitations should be recognized. First, obtaining optimal images is operator dependent. Second, image quality is highly dependent on several patient-related factors, such as the degree of calcification in the aortic annulus or left ventricular outflow tract, which can obscure accurate visualization of the valve and aortic root. Additionally, TTE may be less effective in patients with obesity or in those with suboptimal acoustic windows. The evaluation of the mid-ascending aorta and aortic arch remains challenging due to poor acoustic windows in these regions. Furthermore, while TTE can identify abnormalities in the proximal aorta, it does not offer a continuous or comprehensive assessment of the entire aorta, particularly in the mid to distal regions of the ascending aorta, where blind spots are common. Thus, while TTE serves as a valuable screening tool, it is often necessary to pursue complementary imaging modalities for more detailed evaluation if any abnormalities are detected.

### 3.2. Transesophageal Echocardiography (TEE)

Transesophageal echocardiography (TEE) offers valuable alternative to transthoracic echocardiography (TTE) for the assessment of the AV and aortic root. Due to the anatomical proximity of the esophagus to the aorta, TEE typically provides superior image quality compared to TTE, particularly in patients with suboptimal acoustic windows or poor-quality TTE images. Furthermore, the TEE probe can be manipulated within the esophagus, allowing for the detailed visualization of nearly all segments of the aorta, from the aortic root to its insertion into the diaphragm. From a mid-esophageal position, a short-axis view of the AV can be obtained, typically with the ultrasound beam angled at 45 degrees. Adjusting the angle to 110–140 degrees provides a long-axis view of the AV, which is ideal for obtaining precise measurements of aortic diameters, including those of the sinuses of Valsalva, the sinotubular junction and the proximal ascending aorta. With the slight cranial withdrawal of the probe, combined with a reduction in the angle by approximately 10 degrees and anteflexion of the probe, visualization of the mid-ascending aorta can also be achieved. For assessment of the descending aorta, the probe is rotated to the left at the mid-esophageal position while moving the probe in the cranio-caudal direction, allowing for the visualization of the descending aorta in its entirety, from the distal aortic arch to the diaphragm. This approach provides comprehensive imaging of the descending aorta. To assess the distal aortic arch, the probe is rotated to the right once the junction of the descending aorta and aortic arch is visualized. It is important to note, however, that due to the presence of air in the trachea, TEE does not always allow for optimal visualization of the distal ascending aorta and the proximal arch. While TEE is generally a safe and effective imaging modality, it is more invasive than the other available imaging techniques, and careful consideration should be given for its use in specific patient populations.

### 3.3. Computed Tomography (CT)

Computed tomography (CT) of the chest has emerged as the gold-standard imaging modality for the comprehensive evaluation of aortic aneurysms due to its superior spatial and temporal resolution, widespread availability, rapid acquisition times and ability for three-dimensional reconstruction of the images. CT allows for the assessment of the entire aorta, from the aortic root to the descending thoracic aorta, and can be extended to evaluate the abdominal aorta and its major branches.

**Figure 2:**
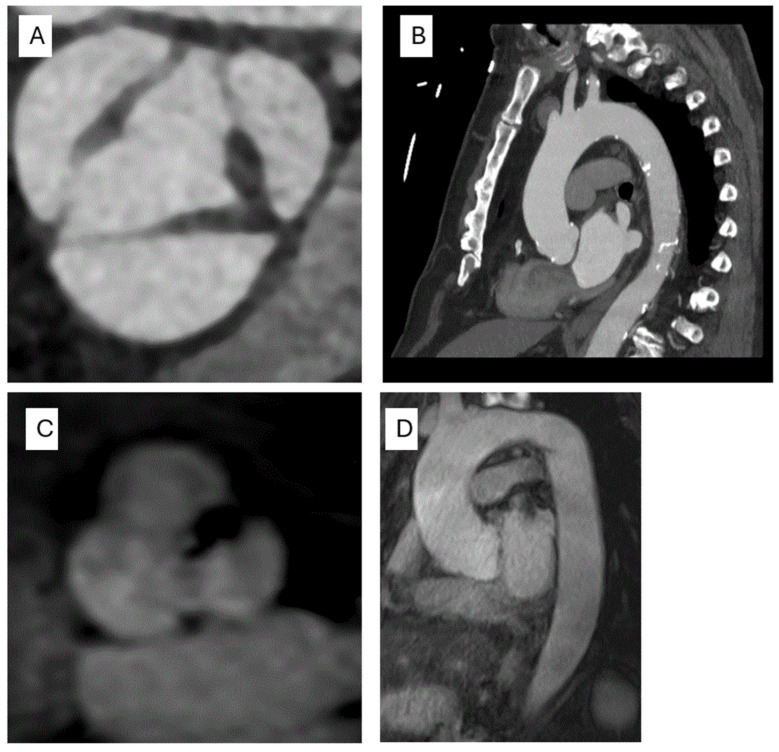
Evaluation of the aortic valve and thoracic aorta by CTA and CMR. (**A**) Short axis of the aortic valve reconstructed to mimic a short-axis view demonstrating BAV with right and left coronary cusps and a raphe at the commissure. (**B**) Visualization of the entire thoracic aorta using MPR. (**C**) Short axis of the aortic valve suggesting the fusion of the right and left coronary cusps. (**D**) CMR allows for the evaluation of the entire aorta and offers MPR. CMR: Cardiac Magnetic Resonance; BAV: Bicuspid Aortic Valve; MPR: Multi-planar Reconstruction; CTA: Computed Tomography Angiography.

For optimal imaging of the aortic root, electrocardiogram (ECG) gating is commonly employed to synchronize the acquisition with end-diastole, minimizing motion artifacts caused by the pulsatile nature of the aorta. While there is no universally accepted method for measuring aortic diameter, at our institution, we typically utilize multi-planar reconstruction (MPR) to generate axial images at key aortic levels, including the sinuses of Valsalva, proximal ascending aorta, aortic arch and descending aorta. These images are then analyzed with a region of interest to measure the maximal aortic diameter at each respective level TEE (Figure 2A,B). The two most common techniques for measuring aortic diameter are the double oblique view and curvilinear reconstruction along the center line.

CT also offers a high-resolution visualization of AV leaflet morphology. Furthermore, aortic regurgitation (AR) can be assessed, though the severity and underlying mechanisms of AR are more reliably determined by transesophageal echocardiography (TEE).

It is important to note that CT imaging involves exposure to ionizing radiation and potential side effects from intravenous contrast agents. However, recent advancements in CT technology have significantly reduced radiation doses. Non-contrast CT can often yield accurate aortic measurements, making it a useful modality for both initial evaluation and longitudinal surveillance. Although its sensitivity for detecting aortic wall abnormalities and acute aortic pathology is lower than that of contrast-enhanced CT, non-contrast CT maintains high accuracy for dimensional assessment when ECG gating is utilized.

### 3.4. Cardiac Magnetic Resonance (CMR)

Magnetic resonance imaging (MRI) offers a valuable alternative to CT imaging in the assessment of aortopathy. While MRI has lower spatial and temporal resolution compared to CT, it eliminates the risks associated with ionizing radiation and iodinated contrast agents, making it particularly suitable for younger patients requiring long-term follow-up, as well as pregnant patients. Like CT, MRI enables the multi-planar reconstruction (MPR) and comprehensive visualization of the entire aorta and EKG gating. It has the added advantage of being able to assess cardiac function, flow quantification and valvular abnormalities. Moreover, 4-D CMR techniques allow for the assessment of time-resolved blood velocity and wall shear stress within the aorta. At our institution, we routinely perform MR angiography without the need for gadolinium-based contrast agents. However, image quality can be compromised in patients with arrhythmias or implanted devices (Figure 2C,D).

## 4. Diagnostic Approach

**Figure 3:**
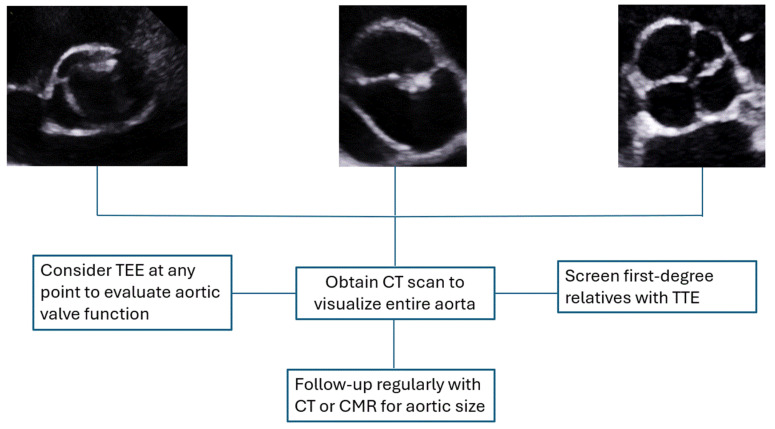
Proposed algorithm in utilizing multimodality imaging in the evaluation of abnormal aortic valve morphology and aortic aneurysms. TTE: Transthoracic Echocardiography; TEE: Transesophageal Echocardiography; CT: Computed Tomography; CMR: Cardiac Magnetic Resonance.

Figure 3 shows a proposed algorithm to help select the appropriate imaging modality in patients with AV dysfunction and aortopathy. TTE is often the first modality in the evaluation of aortic aneurysm and AV dysfunction. If screening with TTE is suggestive of aortic dilation in the visualized portions of the aorta, we recommend proceeding with CT aorta to evaluate the entire length of the aorta. Serial scanning of TAA can be achieved with either non-contrast CT or CMR. CMR can also be considered the test of choice after TTE in patients with pregnancy or kidney dysfunction.

### 4.1. Surveillance

There is substantial evidence in the literature indicating that increased aortic size is associated with a higher risk of aortic dissection and mortality [34]. Consequently, the early diagnosis of aortic aneurysm and a well-defined surveillance strategy are essential for optimal management. Screening first-degree relatives of patients with non-tricuspid AV is also recommended.

Although the rate of aortic growth varies among individuals, it is estimated that in patients with a tricuspid AV, the average growth rates of the ascending aorta, aortic arch and descending aorta are 0.1 mm/year, 0.25 mm/year and 0.19 mm/year, respectively [35,36]. Abnormalities of the AV are associated with a modest increase in the rate of aortic aneurysm progression. Ascending aortic aneurysms in patients with bicuspid AV (BAV) grow at an average rate of 0.3 mm/year, while those with the higher-risk root phenotype have a growth rate that is approximately double that of the general population [35,37].

Once TAAs are detected, imaging of the entire aorta, preferably with CTA, is recommended, as is the evaluation of AV function. Regular surveillance of aortic dimensions is strongly recommended and can be accomplished using any of the aforementioned imaging modalities. The selection of the optimal modality is influenced by multiple factors, including aneurysm size and location, concurrent AV pathology, patient body habitus, image quality from prior studies, renal function, institutional imaging resources and local expertise.

In patients with BAV, a structured lifelong monitoring strategy for thoracic aortic aneurysm (TAA) is warranted if the aortic diameter exceeds 4.0 cm at any level. The frequency of follow-up imaging is dictated by both absolute aortic size and growth rate. In most cases, annual surveillance is appropriate to assess disease progression. However, in patients with TAA exceeding 5.0 cm, more frequent monitoring is advisable. While no established guidelines exist for surveillance intervals in patients with UAV or QAV, a similar individualized approach based on clinical judgment is recommended [2,38].

### 4.2. Medical Management

Subsequent management aims to prevent further increases in the size of the aneurysm and aortic dissection. This is achieved by a combination of blood pressure control, lifestyle modification and smoking cessation. Specific blood pressure medications that might be specifically beneficial in patients with TAAs are angiotensin-receptor blockers and beta blockers, based on observational studies [39]. These medications are thought to attenuate the potential adverse impact of proteolytic enzymes on medial degeneration and help decrease sheer stress on the aortic wall. Statin medications can be prescribed as per the general recommendations for lipid-lowering guidelines [40].

### 4.3. Surgical Intervention

For asymptomatic patients with BAV, guidelines continue to recommend an aneurysm size cutoff of >5.5 cm as a reference for elective surgical repair. However, patients with BAV exhibiting a “root phenotype”, a family history of aortic dissection, an annual aneurysm growth rate exceeding 0.3 cm/year, or concomitant coarctation of the aorta are considered at higher risk and should be referred for surgery at a lower aortic size, typically between 5.0 and 5.4 cm [35]. Other gene-positive TAAs warrant variable surgical thresholds based on the specific mutation involved. For instance, individuals with mutations in the *fibrillin-1* (*FBN1*) gene—commonly associated with Marfan syndrome—are at an elevated risk for aortic dissection. Consequently, elective surgical repair is advised at a reduced aortic diameter of 4.5–5.0 cm in this population [2]. Similarly, pathogenic variants in transforming growth factor-beta (*TGF-β*) genes, as seen in LDS, are associated with accelerated aortic growth and increased mortality. As a result, earlier intervention is recommended, with surgical thresholds ranging from 4.0 to 5.0 cm depending on the specific mutation identified [41].

One of the challenges in determining the optimal cutoff for surgical intervention lies in the fact that aortic dissections can occur even at smaller aortic diameters. Svensson et al. emphasized the importance of indexing aortic cross-sectional area to the patient’s height in predicting adverse outcomes. Their findings indicate that dissections tend to occur at smaller diameters in shorter individuals [42]. At our institution, we prefer to report the aortic root and ascending aortic cross-sectional area in patients with BAV and calculate the cross-sectional area (cm^2^)-to-height (m) ratio. This approach is supported as a Level 2a recommendation in the 2022 American College of Cardiology/American Heart Association Guidelines for the Diagnosis and Management of Aortic Disease [2,43]. Aortic dissection in patients with QAV has not been reported. However, these patients often require surgery for aortic regurgitation and should be followed up closely for the development of this entity. Similarly, there is no defined aortic size cutoff for surgical intervention in patients with UAV. Close follow-up is warranted because of the high risk of aortic stenosis. Mookaddam et al. reported that in a cohort of 232 patients with UAV, 82% of the patients underwent surgery due to isolated AV dysfunction alone without the need for aortic surgery, while aortic surgery for concomitant TAA was present in 23% of the patients necessitating a Bentall-type procedure [15].

Surgical intervention is a Class I recommendation for TAA exceeding 5.5 cm. Two primary approaches exist for TAA management: open surgical repair (OSR) and endovascular stent grafting [44]. OSR involves the excision of the aneurysmal segment and replacement with a prosthetic graft, which is then anastomosed to the non-aneurysmal aorta. This approach is preferred for aneurysms involving the aortic root, ascending aorta or aortic arch. When AV function remains intact, valve-sparing techniques should be pursued whenever feasible [2]. Conversely, endovascular stent grafting aims to exclude blood flow from the aneurysmal sac, thereby reducing the risk of rupture. This technique is FDA-approved for aneurysms involving the descending, juxtarenal and infrarenal aorta [45]. Hybrid repair (HR), which combines open surgical techniques with endovascular stent placement, has emerged as a viable alternative for high-risk patients who cannot tolerate cardiopulmonary bypass, hypothermic circulatory arrest or prolonged aortic cross-clamping—components integral to conventional open surgical repair (OSR) [46]. The primary objective of HR is to minimize the invasiveness associated with OSR [47]. This approach can be performed as a single-stage or staged (two-step) procedure.

One of the main challenges in endovascular repair of the aortic arch is the presence of the supra-aortic branches—the brachiocephalic, left common carotid and left subclavian arteries. To address this, HR typically begins with open surgical debranching and revascularization of these vessels, followed by endovascular stent-graft deployment in the arch, commonly referred to as the frozen elephant trunk (FET) technique. In patients with extensive aortic pathology extending beyond the arch (mega aorta), the endovascular component can be extended to include the descending thoracic and thoracoabdominal aorta [48,49]. Ongoing advancements in device engineering and surgical methodology continue to refine these techniques. For instance, modifications to the FET approach now incorporate fenestrated grafts at customizable locations. Additionally, newer stent-graft designs utilizing advanced biomaterials are being developed, aiming to improve vessel compliance and promote favorable aortic remodeling. As these innovations progress, further research will be essential to assess their clinical benefits, limitations and comparative outcomes relative to established approaches [49].

Following successful repair, lifelong surveillance with annual serial imaging is recommended to monitor for potential complications, including residual aneurysm formation, pseudoaneurysm, endoleaks and graft thrombosis. CTA remains the preferred non-invasive modality for postoperative monitoring.

## 5. Conclusions

In conclusion, abnormal AV and aortopathy are intricate, multifactorial disorders that require a comprehensive approach. Early detection through advanced multimodality imaging is crucial for accurate diagnosis and monitoring. Management strategies should prioritize regular surveillance to minimize the risk of life-threatening complications such as rupture or dissection. Although most cases of non-trileaflet AV seem to be sporadic, more genetic studies are required to delineate the genetic components that could be implicated in the development of these entities.

## Figures and Tables

**Table 1:** Standard multimodality cardiac imaging techniques and protocols for evaluating diseases of the aorta and aortic valve.

	TTE	TEE	CT	CMR
Indication				
Techniques for assessing the aorta	(a) 2-D and 3-D echocardiography (b) Doppler echocardiography	(a) 2-D and 3-D echocardiography (b) Doppler echocardiography	(a) EKG-gated imaging of TAA ± abdomen/pelvis imaging (b) Imaging can be performed with or without IV contrast (c) Axial imaging with MPR (d) For AV morphology, 4-D retrospective gating is recommended	(a) Non-contrast magnetic resonance angiography (MRA) with whole-heart sequence or MRA with gadolinium contrast (b) Flow quantification sequences for hemodynamics in the aortic root for AR evaluation (c) MPR
Techniques for assessing the AV	(a) 2-D and 3-D echocardiography for AV anatomy evaluation (b) Color Doppler for the evaluation of aortic regurgitation (vena contracta, regurgitant orifice area) (c) Continuous-wave Doppler to evaluate gradients across the AV (d) Planimetry for AV area	(a) 2-D and 3-D echocardiography for AV anatomy evaluation (b) Color Doppler for the evaluation of aortic regurgitation (vena contracta, regurgitant orifice area) (c) Continuous-wave Doppler to evaluate gradients across the AV (d) Planimetry for AV area	(a) 4-D retrospective gating (b) MPR for annulus dimensions for AV replacement planning	(a) Dynamic cine imaging for AV analysis (b) Flow quantification sequences for hemodynamics in the aortic root for aortic regurgitation evaluation

2-D: Two dimensional; 3-D: Three dimensional; TAA: Thoracic aortic aneurysm; AV: Aortic Valve; TTE: Transthoracic Echocardiography; TEE: Transesophageal Echocardiography; CT: Computed Tomography; CMR: Cardiac Magnetic Resonance.

**Table 2:** Strengths and limitations of cardiac imaging modalities for aortic valve and aortic aneurysm evaluation.

	TTE	TEE	CT	CMR
Strengths	Portability Wide availability Safety profile Lower cost High temporal resolution Excellent visualization of the aortic root and the proximal ascending aorta Assessment of AV function	High spatial and temporal resolution Superior image quality compared to TTE Allows for better visualization of the aortic root and most of the ascending aorta and arch compared to TTE Best modality to evaluate AV function	High availability Short acquisition time Gold standard High spatial resolution Evaluates AV morphology and the entire length of the aorta MPR Arch branch vessel evaluation Preprocedural planning Can evaluate chamber size and function	High spatial resolution Evaluates AV morphology and the entire length of the aorta (alternative to CT) Preprocedural planning Chamber size and function (gold standard)
Limitations	Operator and body habitus dependent Incomplete evaluation of the aortic arch, ascending and descending aorta compared to other modalities	More invasive than the other modalities Requires sedation Inability to evaluate the distal ascending aorta and proximal arch	Nonportable Iodinated contrast (avoid in renal failure) Ionizing radiation Prone to artifacts (especially if gating is not used)	Lower spatial resolution compared to CT and echo? Lower temporal resolution Lower availability Longer study Gadolinium contrast Artifacts are common with implantable devices Claustrophobia

AV: Aortic Valve; TTE: Transthoracic Echocardiography; TEE: Transesophageal Echocardiography; CT: Computed Tomography; CMR: Cardiac Magnetic Resonance; MPR: Multi-planar Reconstruction.

## Data Availability

No new data were created or analyzed in this study. Data sharing is not applicable to this article.
